# Effect of SGLT2 inhibitors on liver fat content: A meta-analysis

**DOI:** 10.17305/bb.2025.12203

**Published:** 2025-04-25

**Authors:** Quanli Ge, Fengling Zhang, Yong Liu

**Affiliations:** 1 Department of Pharmacy, Yanta Ishan Hospital, Yantai, China; 2 Department of Pharmacy, Yantai Beihai Hospital, Yantai, China

**Keywords:** Sodium-glucose co-transporter-2, SGLT2, liver fat content, LFC, type 2 diabetes, T2DM

## Abstract

Metabolic dysfunction-associated steatotic liver disease (MASLD) is a major metabolic disorder linked to increased morbidity and mortality. Sodium-glucose co-transporter-2 (SGLT2) inhibitors, commonly used to manage type 2 diabetes (T2DM), have shown potential in reducing liver fat content (LFC). However, the magnitude and consistency of this effect remain uncertain. This meta-analysis aimed to evaluate the impact of SGLT2 inhibitors on LFC in adults with metabolic disorders. A systematic search of PubMed, Embase, the Cochrane Library, and Web of Science was conducted up to January 2, 2024, to identify randomized controlled trials (RCTs) assessing the effects of SGLT2 inhibitors on LFC. Studies were included if they reported liver fat changes measured by magnetic resonance imaging-derived proton density fat fraction (MRI-PDFF) or proton magnetic resonance spectroscopy (^1^H-MRS). We pooled standardized mean differences (SMDs) and 95% confidence intervals (CIs) using a random-effects model to account for variability across studies. Thirteen RCTs with 14 datasets (*n* ═ 791 participants) were included. SGLT2 inhibitors significantly reduced LFC compared to controls (SMD: −0.73, 95% CI: −0.97 to −0.50; *P* < 0.001), with moderate heterogeneity (*I*^2^ ═ 62%). Subgroup and meta-regression analyses did not identify any study characteristics—such as study design, diabetic status, patient demographics, baseline LFC, type of SGLT2 inhibitor, or treatment duration—as significant contributors to heterogeneity (all *P* > 0.05). In conclusion, SGLT2 inhibitors are associated with a significant reduction in LFC in adults, supporting their potential role in managing MASLD.

## Introduction

Non-alcoholic fatty liver disease (NAFLD) is a prevalent metabolic disorder, affecting approximately 32% of the global population [1, 2]. It encompasses a spectrum of liver conditions characterized by excessive hepatic fat accumulation in the absence of significant alcohol consumption [3]. Recently, the terminology has shifted to metabolic dysfunction-associated steatotic liver disease (MASLD), which more accurately reflects the metabolic risk factors underlying the disease [4]. Although NAFLD and MASLD share similar diagnostic criteria, MASLD places greater emphasis on the role of metabolic dysfunction—such as obesity, type 2 diabetes (T2D), and dyslipidemia—in disease progression [5]. Both conditions increase the risk of non-alcoholic steatohepatitis (NASH), liver fibrosis, cirrhosis, hepatocellular carcinoma, and cardiovascular complications, contributing significantly to morbidity and mortality [6]. Liver fat content (LFC) is a key pathological feature of NAFLD/MASLD and plays a crucial role in disease progression [7]. Excess hepatic lipid accumulation promotes insulin resistance, hepatic inflammation, and fibrosis, which are central to the pathogenesis of NAFLD/MASLD [8]. Consequently, reducing LFC is a major therapeutic goal aimed at mitigating disease progression and its associated metabolic complications [9]. LFC is assessed using various imaging techniques and histological evaluations [10]. While liver biopsy remains the diagnostic gold standard, its invasive nature limits its routine clinical use [10]. Non-invasive imaging modalities, such as magnetic resonance imaging-derived proton density fat fraction (MRI-PDFF) and proton magnetic resonance spectroscopy (^1^H-MRS) have emerged as highly accurate and reproducible methods for quantifying LFC [11–13]. Compared to ultrasound-based techniques, MRI-based methods provide superior sensitivity and allow for precise quantification and longitudinal monitoring of LFC changes in response to treatment [14]. Despite increasing recognition of NAFLD/MASLD as a major health concern, no pharmacological therapies have been approved specifically to reduce LFC [15]. Current evidence-based management primarily focuses on lifestyle interventions—particularly weight loss through diet and exercise—which have been shown to improve hepatic steatosis and insulin sensitivity [16]. However, long-term adherence to lifestyle changes is often difficult, underscoring the need for targeted pharmacological treatments. Sodium-glucose co-transporter-2 (SGLT2) inhibitors, a class of antidiabetic agents, lower blood glucose levels by promoting urinary glucose excretion [17]. Originally developed for managing T2D, these agents also confer metabolic and cardiovascular benefits beyond glycemic control [18, 19]. SGLT2 inhibitors promote weight loss, enhance insulin sensitivity, reduce blood pressure, and offer protective effects on the cardiovascular and renal systems [20]. Emerging evidence suggests that SGLT2 inhibitors may also affect hepatic lipid metabolism, making them a promising therapeutic option for reducing LFC in patients with NAFLD/MASLD [21]. However, the impact of SGLT2 inhibitors on hepatic fat accumulation varies across studies: some report significant reductions in LFC [22–32], while others show minimal or inconsistent effects [33, 34]. Therefore, in this study, we conducted a meta-analysis of randomized controlled trials (RCTs) to systematically evaluate the effects of SGLT2 inhibitors on LFC in adults with metabolic disorders, using MRI-based assessments.

## Materials and methods

This meta-analysis was designed and conducted in accordance with the Preferred Reporting Items for Systematic Reviews and Meta-Analyses (PRISMA) 2020 guidelines [35, 36] and the Cochrane Handbook for Systematic Reviews of Interventions [37]. The study protocol was prospectively registered in PROSPERO under the identifier CRD42025632495.

### Search strategy

A systematic search was conducted across PubMed, Embase, the Cochrane Library, and Web of Science databases from their inception to January 2, 2024. The search strategy combined terms related to: (1) “sodium-glucose transporter 2 inhibitor” OR “sodium-glucose transporter II inhibitor” OR “SGLT2 inhibitor” OR “SGLT-2 inhibitor” OR “SGLT2” OR “sodium-glucose cotransporter 2 inhibitors” OR specific drug names, including “canagliflozin,” “dapagliflozin,” “empagliflozin,” “ertugliflozin,” “tofogliflozin,” “bexagliflozin,” “henagliflozin,” “ipragliflozin,” “licogliflozin,” “luseogliflozin,” “remogliflozin,” “sergliflozin,” and “sotagliflozin”; (2) “liver” OR “hepatic”; (3) “fat,” “adipose,” “adiposity,” OR “lipid”; and (4) “random,” “randomly,” “randomized,” “control,” “allocated,” “placebo,” “controls,” OR “RCT.” The detailed search strategy for each database is provided in Supplemental data. Only full-length articles published in peer-reviewed journals and written in English were included. Additionally, the reference lists of relevant articles and reviews were manually screened to identify further studies. Duplicate records were removed using EndNote X4 (Thomson Reuters, New York, NY, USA) reference management software.

### Inclusion and exclusion criteria

We included studies that met the following criteria, which were designated according to the Population, Intervention, Comparison, Outcomes and Study (PICOS) principle [38].

Population (P): Adults with metabolic disorders or conditions associated with NAFLD or MASLD, including but not limited to obesity, T2D, and metabolic syndrome.

Intervention (I): SGLT2 inhibitors on the basis of background treatments (such as other concurrent anti-diabetic medications for patients with T2D) for at least one week.

Control (C): Placebo or no additional treatments on the basis of background treatments.

Outcomes: The difference for the changes of LFC after treatment between patients allocated to the intervention and control groups, which was evaluated by MRI-based methods, such as MRI-PDFF or ^1^H-MRS.

Study design: RCTs, including cross-over studies and parallel-group RCTs.

Reviews, case reports, editorials, animal studies, and observational studies were excluded. Studies involving pediatric populations were also excluded. Additionally, we excluded studies in which SGLT2 inhibitors were administered for less than seven days, as our goal was to avoid assessing the acute effects of these medications on LFC. Studies comparing SGLT2 inhibitors with active controls or those that did not report the outcome of interest were also excluded. In cases of overlapping study populations, the study with the largest sample size was selected for inclusion in the meta-analysis.

### Data extraction

Two independent reviewers screened the titles, abstracts, and full texts of the studies, and extracted relevant data using a standardized data extraction form. Any discrepancies were resolved through discussion or by consulting a third reviewer. The extracted data included study characteristics (author, year of publication, country, and study design), participant characteristics (diagnosis, sample size, mean age, proportion of male participants, baseline HbA1c, body mass index [BMI], and diabetes duration), methods used to evaluate LFC, baseline LFC values, concurrent anti-diabetic treatments, specific SGLT2 inhibitors and their dosages, control group details, and treatment durations.

### Quality assessment

The quality of the included studies was assessed using the Cochrane Risk of Bias Tool, which evaluates potential sources of bias across multiple domains [39]. These domains include selection bias (random sequence generation and allocation concealment), performance bias (blinding of participants and personnel), detection bias (blinding of outcome assessors), attrition bias (incomplete outcome data), reporting bias (selective reporting), and other sources of bias. Each study was rated as having a low, high, or unclear risk of bias in each domain [39]. Discrepancies were resolved through discussion or, when necessary, consultation with a third reviewer. A risk-of-bias summary table was generated to visualize the assessment results. In addition, the certainty of evidence was assessed using the Grading of Recommendations Assessment, Development and Evaluation (GRADE) system, which considers factors, such as risk of bias, inconsistency, indirectness, imprecision, and publication bias [40]. Based on these criteria, the certainty of evidence was classified as very low, low, moderate, or high.

**Figure 1.**
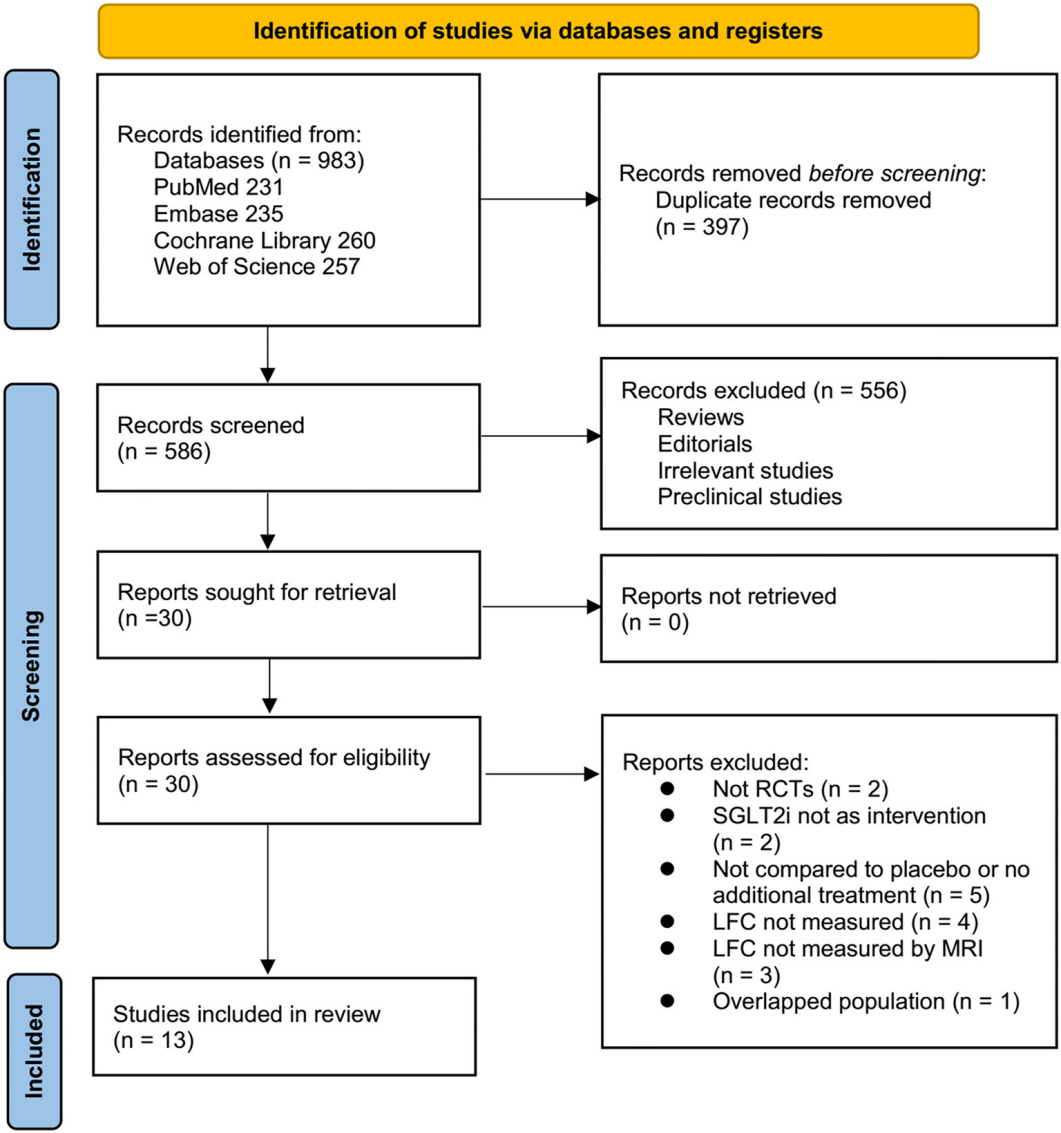
**Flowchart of study inclusion**. LFC: Liver fat content; SGLT2: Sodium-glucose co-transporter-2; RCT: Randomized controlled trial.

### Statistical analysis

The differences in changes in LFC after treatment between patients allocated to SGLT2 inhibitors and those in the control groups were summarized using standardized mean differences (SMDs) with corresponding 95% confidence intervals (CIs) [37], as different MRI-based methods were used to assess LFC across the included studies. Between-study heterogeneity was evaluated using the Cochrane *Q* test [37], while the extent of heterogeneity was assessed with the *I*^2^ statistic. Values of *I*^2^ <25%, 25%–75%, and >75% were considered to indicate mild, moderate, and substantial heterogeneity, respectively [41]. Meta-analysis was conducted using the inverse variance (IV) method with a random-effects model to account for potential heterogeneity among studies [37]. Sensitivity analyses were performed by excluding one dataset at a time to assess the robustness of the findings [42]. Subgroup analyses were conducted to explore the impact of predefined study characteristics on outcomes, including study location (Asian vs non-Asian), study design (double-blind vs open-label), participants’ diabetic status, mean age, proportion of male participants, baseline HbA1c, baseline BMI, methods used for LFC measurement, baseline LFC, type of SGLT2 inhibitor used, and treatment duration. Medians of continuous variables were used as cutoffs for subgroup classification. In addition, a univariate meta-regression analysis was performed to examine the influence of continuous study characteristics—such as sample size, mean age, proportion of men, baseline HbA1c, BMI, LFC, and treatment duration—on the meta-analysis results. Potential publication bias was assessed using funnel plots, and Egger’s regression test was used to detect small-study effects [43]. A *P* value <0.05 in Egger’s test was considered indicative of publication bias. All statistical analyses were conducted using RevMan (version 5.1; Cochrane Collaboration, Oxford, UK) and Stata (version 17.0; Stata Corporation, College Station, TX, USA).

## Results

### Literature search and study identification

A detailed PRISMA flow diagram is provided in Figure 1. The initial search identified 983 records across four databases. After removing 397 duplicates, 586 unique articles remained. Title and abstract screening led to the exclusion of 556 studies, primarily due to irrelevance to the meta-analysis objective. Of the 30 studies assessed in full-text review, 17 were excluded for reasons detailed in Figure 1. Ultimately, 13 RCTs [22–34] met the inclusion criteria and were included in the meta-analysis.

### Study characteristics

A summary of the study characteristics is presented in Table 1. In total, 13 RCTs were included in the meta-analysis, comprising two cross-over studies [29, 34] and 11 parallel-group studies [22–28, 30–33]. One study [31] reported two separate datasets for participants with and without diabetes; these were analyzed independently, resulting in 14 datasets overall. The included studies were published between 2018 and 2024 and were conducted in a range of countries, including India, Sweden, Finland, the United States, Germany, France, Japan, Egypt, the United Kingdom, China, and the Netherlands. Four datasets included patients with T2D and NAFLD [22, 23, 27, 30]; seven datasets included patients with T2D only [24–26, 28, 29, 31, 33]; one included pre-diabetic participants [34]; and the remaining two included non-diabetic overweight participants [31] and non-diabetic patients with MASLD [32]. Sample sizes across studies ranged from 14 to 160 participants. Mean ages varied from 45.0 to 66.3 years, and the proportion of male participants ranged from 39.2% to 80.6%. Baseline mean HbA1c levels ranged from 5.5% to 9.1% and baseline mean BMI ranged from 27.4 to 35.2 kg/m^2^. LFC was assessed using MRI-PDFF in six studies [22, 23, 25, 27, 30, 32], and ^1^H-MRS in the remaining seven studies [24, 26, 28, 29, 31, 33, 34]. Baseline mean LFC ranged from 6.2% to 26.5%. The interventions involved SGLT2 inhibitors: dapagliflozin in six studies [22, 25, 28–30, 34], empagliflozin in six studies [23, 26, 27, 31–33], and canagliflozin in one study [24]. The control groups received either a placebo (11 studies: [22–27, 29, 31–34]) or no additional treatment beyond standard therapy (two studies: [28, 30]). Treatment durations ranged from two to 52 weeks.

**Table 1:** Characteristics of the included RCTs

**Study**	**Country**	**Design**	**Patient diagnosis**	**Patient number**	**Mean age (years)**	**Male (%)**	**Baseline HbA1c (%)**	**Duration of diabetes (years)**	**Baseline BMI (kg/m^2^)**	**Methods of MR-LFC measurement**	**Baseline LFC (%)**	**Concurrent anti-diabetic treatment**	**Intervention**	**Control**	**Treatment duration (weeks)**
Kuchay, 2018	India	R, OL	T2D and NAFLD	42	49.9	59.5	9.1	6.7	29.7	Average MRI-PDFF of nine-segment	16.3	Metformin, DPP-4 inhibitors, SUs, or insulin	Empagliflozin 10 mg/d	No additional treatment	20
Eriksson, 2018	Sweden	R, DB, PC	T2D and NAFLD	38	65.3	78.6	7.4	6.6	30.2	MRI-PDFF covering the entire liver volume	16.2	Metformin, or SUs, 14% were drug naive	Dapagliflozin 10 mg/d	Placebo	12
Latva-Rasku, 2019	Finland	R, DB, PC	T2D	31	60.9	80.6	6.9	7.5	32	Median LFC by MRI-PDFF covering the entire liver volume	21.5	Metformin, or DPP-4 inhibitors	Dapagliflozin 10 mg/d	Placebo	8
Cusi, 2019	USA	R, DB, PC	T2D	51	58	66.1	7.7	NR	31.5	Liver ^1^H-MRS	12.3	Metformin, or DPP-4 inhibitors	Canagliflozin 300 mg/d	Placebo	24
Kahl, 2020	Germany	R, DB, PC	T2D	84	62.1	69	6.6	3.3	32.2	Liver ^1^H-MRS	10.4	None	Empagliflozin 25 mg/d	Placebo	24
Gaborit, 2021	France	R, DB, PC	T2D	51	56.9	39.2	8.1	11.1	34.9	Liver ^1^H-MRS	26.5	Metformin, DPP-4 inhibitors, SUs, or insulin	Empagliflozin 10 mg/d	Placebo	12
Horibe, 2022	Japan	R, OL	T2D	43	60.9	64	7.7	12.5	27.8	Liver ^1^H-MRS	22.1	Metformin, DPP-4 inhibitors, SUs, or insulin	Dapagliflozin 5 mg/d	No additional treatment	24
Elhini, 2022	Egypt	R, DB, PC	T2D and NAFLD	160	47.5	65	8.5	NR	32.3	Average MRI-PDFF of nine-segment	20.8	Metformin, or SUs	Empagliflozin 25 mg/d	Placebo	24
Rajeev, 2023	UK	R, DB, PC, CO	T2D	45	57.3	63	7.7	NR	35.2	Liver ^1^H-MRS	19.9	Metformin, or SUs	Dapagliflozin 10 mg/d	Placebo	12
Shi, 2023	China	R, OL	T2D and NAFLD	78	48.2	69.2	8.5	NR	30.7	Average MRI-PDFF of nine-segment	14.2	Metformin	Dapagliflozin 10 mg/d	No additional treatment	24
Veelen, 2023	The Netherlands	R, DB, PC, CO	Prediabetes	14	66.3	57.1	5.5	0	30.3	Liver ^1^H-MRS	6.2	None	Dapagliflozin 10 mg/d	Placebo	2
Abdelgani, 2024 DM	USA	R, DB, PC	T2D	30	55	57	7.6	5.9	32.7	Liver ^1^H-MRS	13.6	Metformin, or SUs	Empagliflozin 25 mg/d	Placebo	12
Abdelgani, 2024 NDM	USA	R, DB, PC	Non-diabetic overweight participants	27	45	52	5.5	0	34.3	Liver ^1^H-MRS	11.8	None	Empagliflozin 25 mg/d	Placebo	12
Cheung, 2024	China	R, DB, PC	Non-diabetic patients with MASLD	97	55.7	55.1	5.7	0	27.4	Average MRI-PDFF of nine-segment	9.6	None	Empagliflozin 10 mg/d	Placebo	52

MASLD: Metabolic dysfunction-associated steatotic liver disease; MRI-PDFF: Magnetic resonance imaging-derived proton density fat fraction; ^1^H-MRS: Proton magnetic resonance spectroscopy; LFC: Liver fat content; T2D: Type 2 diabetes; NAFLD: Non-alcoholic fatty liver disease; BMI: Body mass index.

### Study quality evaluation

The details of study quality evaluation using the Cochrane Risk of Bias Tool are presented in Table 2. Three of the included studies were open-label RCTs [23, 28, 30], while the remaining 10 were double-blind RCTs [22, 24–27, 29, 31–34]. Random sequence generation was adequately reported in nine studies [22–24, 26, 28–30, 32, 33], and allocation concealment was sufficiently addressed in the same nine studies [22, 24–26, 28–30, 32, 33]. No bias related to incomplete outcome data or selective reporting was observed.

**Table 2:** Study quality evaluation via the Cochrane risk of bias tool

**Study**	**Random sequence generation**	**Allocation concealment**	**Blinding of participants**	**Blinding of outcome assessment**	**Incomplete outcome data addressed**	**Selective reporting**	**Other sources of bias**
Kuchay, 2018	Low risk	Unclear	High risk	High risk	Low risk	Low risk	Low risk
Eriksson, 2018	Low risk	Low risk	Low risk	Low risk	Low risk	Low risk	Low risk
Latva-Rasku, 2019	Unclear	Low risk	Low risk	Low risk	Low risk	Low risk	Low risk
Cusi, 2019	Low risk	Low risk	Low risk	Low risk	Low risk	Low risk	Low risk
Kahl, 2020	Low risk	Low risk	Low risk	Low risk	Low risk	Low risk	Low risk
Gaborit, 2021	Low risk	Low risk	Low risk	Low risk	Low risk	Low risk	Low risk
Horibe, 2022	Low risk	Low risk	High risk	High risk	Low risk	Low risk	Low risk
Elhini, 2022	Unclear	Unclear	Low risk	Low risk	Low risk	Low risk	Low risk
Rajeev, 2023	Low risk	Low risk	Low risk	Low risk	Low risk	Low risk	Low risk
Shi, 2023	Low risk	Low risk	High risk	High risk	Low risk	Low risk	Low risk
Veelen, 2023	Unclear	Unclear	Low risk	Low risk	Low risk	Low risk	Low risk
Abdelgani, 2024 DM	Unclear	Unclear	Low risk	Low risk	Low risk	Low risk	Low risk
Abdelgani, 2024 NDM	Unclear	Unclear	Low risk	Low risk	Low risk	Low risk	Low risk
Cheung, 2024	Low risk	Low risk	Low risk	Low risk	Low risk	Low risk	Low risk

### Influence of SGLT2 inhibitors on LFC

**Figure 2.**
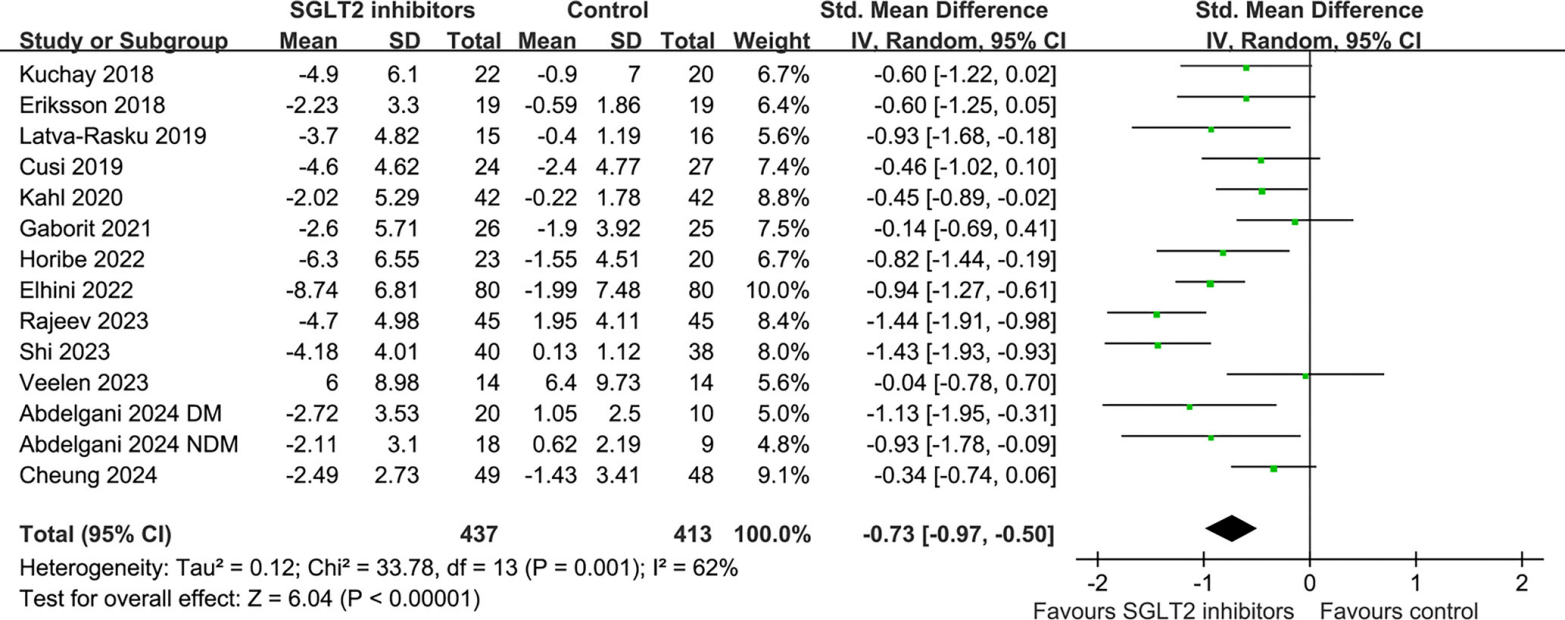
**Forest plots for the meta-analysis comparing the influence between SGLT2 inhibitors with controls on LFC**. LFC: Liver fat content; SGLT2: Sodium-glucose co-transporter-2; CI: Confidence interval; IV: Inverse variance.

Overall, 13 RCTs encompassing 14 datasets [22–34] evaluated the effect of SGLT2 inhibitors on LFC. The pooled results showed that, compared to controls receiving either placebo or no additional treatment, SGLT2 inhibitors significantly reduced LFC in adults (SMD: −0.73, 95% CI: −0.97 to −0.50; *P* < 0.001; Figure 2), with moderate heterogeneity (*I*^2^ ═ 62%). Sensitivity analyses, performed by excluding one dataset at a time, did not materially change the results (SMD range: −0.67 to −0.78; all <0.05). Subgroup analyses indicated that the effect of SGLT2 inhibitors on LFC was not significantly influenced by study country, study design, participants’ diabetic status, mean age, proportion of men, baseline HbA1c, baseline BMI, the method used to measure LFC (MRI-based), specific SGLT2 medications, or treatment duration (all *P* values for subgroup differences >0.05; Table 3). Additionally, univariate meta-regression analyses showed that none of the following variables significantly modified the effect of SGLT2 inhibitors on LFC: study sample size, mean age, proportion of men, baseline HbA1c, BMI, baseline LFC, or treatment duration (all *P* >0.05; Table 4). The certainty of the evidence for LFC outcomes was downgraded by one level (to moderate) due to the statistical heterogeneity observed among the included studies (Table 5).

**Table 3:** Results of subgroup analyses

	**Difference of LFC between patients treated with SGLT2 inhibitors and controls**				
**Variables**	**No. of datasets**	**SMD (95% CI)**	***I* ^2^**	***P* for subgroup effects**	***P* for subgroup difference**
*Study country*					
Asian	4	−0.79 [−1.31, −0.28]	74%	0.003	
Non-Asian	10	−0.71 [−1.00, −0.43]	60%	<0.001	0.79
*Design*					
R, DB, PC	11	−0.67 [−0.93, −0.40]	61%	<0.001	
R, OL	3	−0.98 [−1.50, −0.46]	58%	<0.001	0.29
*Diabetic status*					
T2D	11	−0.82 [−1.07, −0.56]	60%	<0.001	
Non-diabetic	3	−0.38 [−0.77, −0.01]	19%	0.04	0.07
*Mean age (years)*					
<57	7	−0.77 [−1.12, −0.42]	67%	<0.001	
≥57	7	−0.70 [−1.05, −0.35]	61%	<0.001	0.78
*Men (%)*					
<64	7	−0.65 [−1.08, −0.23]	72%	0.003	
≥64	7	−0.81 [−1.08, −0.54]	46%	<0.001	0.54
*Baseline HbA1c (%)*					
<7.7	7	−0.54 [−0.78, −0.30]	11%	<0.001	
≥7.7	7	−0.86 [−1.21, −0.50]	70%	<0.001	0.14
*Baseline BMI (kg/m^2^)*					
<32	7	−0.63 [−0.97, −0.29]	60%	<0.001	
≥32	7	−0.84 [−1.18, −0.49]	64%	<0.001	0.40
*Methods for LFC measuring*					
MRI-PDFF	6	−0.81 [−1.14, −0.48]	61%	<0.001	
^1^H-MRS	8	−0.67 [−1.03, −0.31]	65%	<0.001	0.59
*Baseline LFC (%)*					
<15	7	−0.67 [−1.03, −0.30]	65%	<0.001	
≥15	7	−0.80 [−1.12, −0.49]	58%	<0.001	0.58
*SGLT2 inhibitors*					
Empagliflozin	7	−0.60 [−0.87, −0.34]	46%	0.09	
Dapagliflozin	6	−0.93 [−1.35, −0.50]	66%	0.01	0.21
*Treatment durations (weeks)*					
<24	8	−0.73 [−1.11, −0.35]	62%	<0.001	
≥24	6	−0.74 [−1.06, −0.41]	67%	<0.001	0.98

SMD: Standardized mean difference; CI: Confidence interval; SGLT2: Sodium-glucose co-transporter-2; LFC: Liver fat content; MRI-PDFF: Magnetic resonance imaging-derived proton density fat fraction; ^1^H-MRS: Proton magnetic resonance spectroscopy; BMI: Body mass index; T2D: Type 2 diabetes.

**Table 4:** Results of univariate meta-regression analysis

**Variables**	**SMD for the changes of LFC between patients treated with SGLT2 inhibitors and controls**		
	**Coefficient**	**95% CI**	***P* values**
Sample size	−0.00095	−0.00806 to 0.00616	0.77
Mean age (years)	0.032	−0.008 to 0.072	0.11
Men (%)	−0.017	−0.042 to 0.008	0.18
Baseline HbA1c (%)	−0.16	−0.39 to 0.07	0.15
Baseline BMI (kg/m^2^)	−0.052	−0.166 to 0.062	0.34
Baseline LFC (%)	−0.020	−0.068 to 0.029	0.40
Treatment duration (weeks)	0.0050	−0.0176 to 0.0277	0.64

LFC: Liver fat content; SMD: Standardized mean difference; SGLT2: Sodium-glucose co-transporter-2; BMI: Body mass index; CI: Confidence interval.

**Table 5:** Summarized certainty of evidence using the GRADE system

**Outcome**	**Quality assessment**							**Absolute effect** **SMD (95% CI)**	**Quality**
	**No. of datasets**	**Design**	**Risk of bias**	**Inconsistency**	**Indirectness**	**Imprecision**	**Other considerations**		
LFC (%)	14	RCTs	No serious risk of bias	Serious inconsistency	No serious indirectness	No serious imprecision	None	−0.73 (−0.97 to −0.50)	⊕⊕⊕O MODERATE

GRADE: Grading of Recommendations Assessment, Development and Evaluation; CI: Confidence interval; RCT: Randomized controlled trial; LFC: Liver fat content; SMD: Standardized mean difference.

### Publication bias

**Figure 3.**
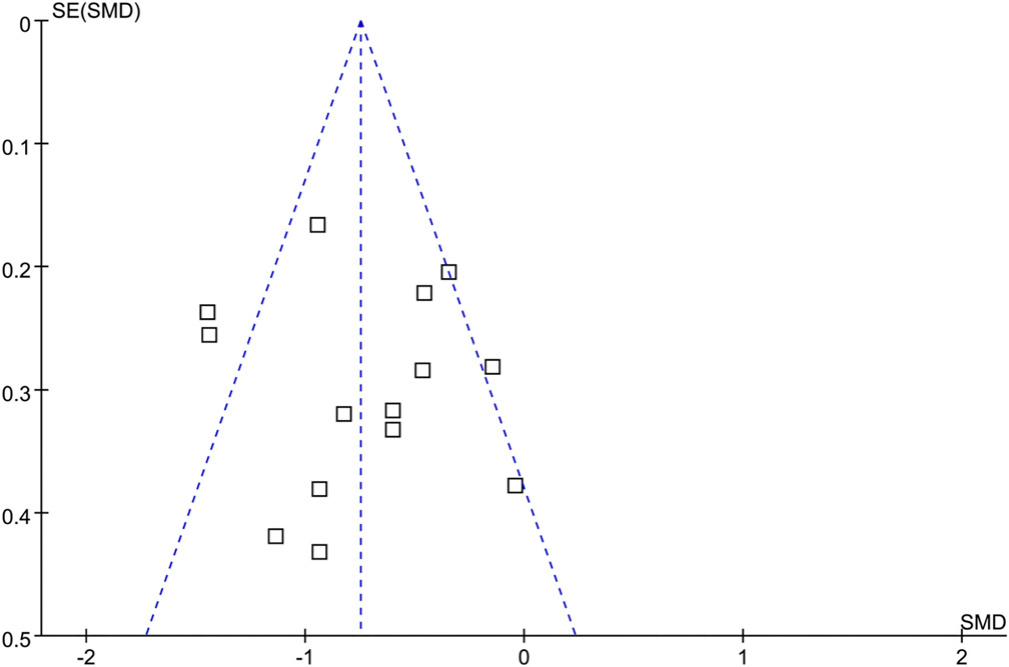
**Funnel plots for the publication bias underlying the meta-analysis comparing the influence between SGLT2 inhibitors with controls on LFC**. LFC: Liver fat content; SGLT2: Sodium-glucose co-transporter-2; SMD: Standardized mean difference.

The funnel plots for the meta-analysis evaluating the influence of SGLT2 inhibitors on LFC are shown in Figure 3. Visual inspection revealed no substantial asymmetry, suggesting a low risk of publication bias. This was further supported by the results of Egger’s regression test, which confirmed the low risk of publication bias (*P* ═ 0.82).

## Discussion

This meta-analysis of 13 RCTs comprising 14 datasets and 791 participants demonstrated that SGLT2 inhibitors significantly reduce LFC in adults with metabolic disorders. Sensitivity analyses confirmed the robustness of these findings, while subgroup and meta-regression analyses did not identify any significant effect modifiers, suggesting that multiple factors may collectively contribute to the observed heterogeneity. These results highlight the potential of SGLT2 inhibitors in managing hepatic steatosis and support their role as a therapeutic option for patients with MASLD. Several mechanisms may underlie the observed reduction in LFC with SGLT2 inhibitors. One proposed pathway is the enhancement of lipid metabolism through increased fatty acid oxidation and reduced hepatic de novo lipogenesis [44]. SGLT2 inhibitors promote lipolysis in adipose tissue and shift energy metabolism toward lipid utilization, thereby decreasing ectopic fat accumulation in the liver [45]. Additionally, these agents improve insulin sensitivity by reducing hyperinsulinemia, which may attenuate insulin-driven lipogenesis and hepatic fat deposition [46]. At the molecular level, SGLT2 inhibitors have been shown to activate AMP-activated protein kinase (AMPK) [47], a key regulator of cellular energy homeostasis that promotes lipid oxidation and inhibits lipogenesis [48]. They may also modulate peroxisome proliferator-activated receptor alpha (PPAR-α) [49], which enhances fatty acid transport and oxidation in the liver [50]. Beyond their metabolic effects, SGLT2 inhibitors exhibit anti-inflammatory properties, reducing both systemic and hepatic inflammation—a critical factor in preventing the progression of MASLD to more advanced stages, such as NASH and fibrosis [51, 52].

Despite the overall positive findings, the presence of moderate heterogeneity (*I*^2^ ═ 62%) suggests variability in treatment response among the included studies. Subgroup and meta-regression analyses did not identify significant sources of heterogeneity, indicating that multiple unmeasured factors may influence the effect of SGLT2 inhibitors on LFC. Potential contributors include differences in dietary intake [53], genetic predisposition [54], and other nutritional or lifestyle factors [55], which were not accounted for at the study level. Individual patient characteristics—such as hepatic insulin resistance, baseline metabolic status, or gut microbiome composition—may also affect responsiveness to SGLT2 inhibitors. Additionally, variations in study design, including treatment adherence, concomitant medications, and imaging techniques used for LFC assessment, could have influenced the results. These findings underscore the complexity of hepatic fat metabolism and highlight the need for further research using individual-level data to identify patient subgroups that may benefit most from SGLT2 inhibitors. This meta-analysis has several strengths. First, it included only RCTs, providing the highest level of evidence regarding the effects of SGLT2 inhibitors on LFC. Second, an extensive and systematic literature search ensured the inclusion of all relevant studies, making this the most comprehensive and up-to-date synthesis of available evidence. Third, the robustness of the findings was confirmed through multiple sensitivity analyses. Furthermore, all included studies utilized MRI-based techniques, which offer more accurate quantification of LFC than ultrasound-based methods, thereby enhancing the reliability of the pooled results. However, several limitations should be acknowledged. The moderate heterogeneity observed limits the certainty of the findings, although no significant sources were identified through subgroup or meta-regression analyses. Additionally, only three SGLT2 inhibitors—dapagliflozin, empagliflozin, and canagliflozin—were evaluated, leaving the effects of other agents in this drug class unclear. Although the subgroup analysis did not reveal significant differences between empagliflozin and dapagliflozin, a comparative analysis for canagliflozin was not feasible due to the inclusion of only one relevant study. Moreover, a dose–response relationship could not be assessed due to insufficient data across different dosage levels. These findings suggest that while SGLT2 inhibitors may share similar mechanisms of action, their relative efficacy in reducing LFC remains uncertain and warrants further investigation through head-to-head RCTs. Furthermore, although subgroup analysis by study region (Asian vs non-Asian) did not reveal significant differences, the lack of individual-level data precluded a direct assessment of the impact of patient ethnicity on treatment outcomes. Ethnic differences in genetic background, dietary patterns, and lifestyle may influence treatment responsiveness. Future patient-level meta-analyses are necessary to determine whether genetic or environmental factors mediate variability in liver fat response among different ethnic groups. It is also noteworthy that none of the included studies enrolled lean individuals, as all reported mean baseline BMI values in the overweight or obese range. Thus, the effect of SGLT2 inhibitors on LFC in lean individuals remains unknown and warrants further study. Another limitation is the treatment duration, which ranged from 2 to 52 weeks. Although a significant reduction in LFC was observed, longer-term studies are needed to evaluate the sustainability of these benefits and whether they translate into improvements in liver histology or clinical outcomes. Finally, as this meta-analysis was based on study-level data, it was not possible to conduct individualized assessments of how patient or study characteristics may influence treatment response. Future large-scale RCTs with individual-level data are essential to better understand the role of baseline metabolic status, genetic factors, and concurrent lifestyle modifications in determining the efficacy of SGLT2 inhibitors in reducing hepatic steatosis.

The clinical implications of these findings are significant. Given the growing burden of MASLD and the lack of approved pharmacological treatments, SGLT2 inhibitors represent a promising option for reducing LFC in patients with metabolic disorders. Their ability to improve multiple metabolic parameters—including glycemic control, body weight, and insulin sensitivity—further supports their role in the comprehensive management of metabolic disease. Future research should investigate whether combining SGLT2 inhibitors with other pharmacological agents, such as GLP-1 receptor agonists or PPAR agonists, yields additive benefits in reducing hepatic fat and preventing MASLD progression. Additionally, long-term studies are needed to evaluate the impact of SGLT2 inhibitors on liver fibrosis, cardiovascular risk, and overall survival to fully establish their therapeutic potential.

## Conclusion

In conclusion, this meta-analysis provides strong evidence that SGLT2 inhibitors significantly reduce LFC in adults with metabolic disorders, highlighting their potential role in the management of MASLD. Although the exact mechanisms are not yet fully understood, the observed benefits are likely mediated by improvements in lipid metabolism, insulin sensitivity, and hepatic inflammation. Given the moderate heterogeneity across studies and the limitations inherent in study-level analyses, further large-scale RCTs with long-term follow-up and patient-level data are warranted to refine treatment strategies and identify the individuals most likely to benefit from SGLT2 inhibitor therapy.

## Supplemental data

**Detailed search strategy for each database**

**PubMed**

(“Sodium-Glucose Transporter 2 Inhibitors”[Mesh] OR “sodium glucose transporter 2 inhibitor” OR “sodium glucose transporter ii inhibitor” OR “SGLT 2 inhibitor” OR “SGLT-2 inhibitor” OR “SGLT2” OR “sodium glucose cotransporter 2 inhibitors” OR “canagliflozin” OR “dapagliflozin” OR “empagliflozin” OR “ertugliflozin” OR “tofogliflozin” OR “bexagliflozin” OR “henagliflozin” OR “ipragliflozin” OR “licogliflozin” OR “luseogliflozin” OR “remogliflozin” OR “sergliflozin” OR “sotagliflozin”) AND (“Randomized Controlled Trial”[Publication Type] OR “randomized” OR “randomly” OR “randomization” OR “placebo” OR “control” OR “allocated” OR “RCT”) AND (“Liver”[Mesh] OR “Hepatic” OR “Liver”) AND (“Fat”[Mesh] OR “adipose” OR “adiposity” OR “lipid”)

**Embase**

(“sodium glucose transporter 2 inhibitor”/exp OR “sodium glucose transporter 2 inhibitor” OR “sodium glucose transporter ii inhibitor” OR “sglt 2 inhibitor” OR “sglt-2 inhibitor” OR “sglt2” OR “sodium glucose cotransporter 2 inhibitors” OR “canagliflozin” OR “dapagliflozin” OR “empagliflozin” OR “ertugliflozin” OR “tofogliflozin” OR “bexagliflozin” OR “henagliflozin” OR “ipragliflozin” OR “licogliflozin” OR “luseogliflozin” OR “remogliflozin” OR “sergliflozin” OR “sotagliflozin”) AND (“randomized controlled trial”/exp OR “randomized controlled trial” OR “randomized” OR “randomly” OR “randomization” OR “placebo” OR “control” OR “allocated” OR “rct”) AND (“liver”/exp OR “hepatic” OR “liver”) AND (“fat”/exp OR “adipose” OR “adiposity” OR “lipid”)

**Cochrane Library**

(“sodium glucose transporter 2 inhibitor” OR “sodium glucose transporter ii inhibitor” OR “SGLT 2 inhibitor” OR “SGLT-2 inhibitor” OR “SGLT2” OR “sodium glucose cotransporter 2 inhibitors” OR “canagliflozin” OR “dapagliflozin” OR “empagliflozin” OR “ertugliflozin” OR “tofogliflozin” OR “bexagliflozin” OR “henagliflozin” OR “ipragliflozin” OR “licogliflozin” OR “luseogliflozin” OR “remogliflozin” OR “sergliflozin” OR “sotagliflozin”) AND (“randomized controlled trial” OR “randomized” OR “randomly” OR “placebo” OR “control” OR “allocated” OR “RCT”) AND (“liver” OR “hepatic”) AND (“fat” OR “adipose” OR “adiposity” OR “lipid”) IN Cochrane Central Register of Controlled Trials (CENTRAL)

**Web of Science**

TS ═ (“sodium glucose transporter 2 inhibitor” OR “sodium glucose transporter ii inhibitor” OR “SGLT 2 inhibitor” OR “SGLT-2 inhibitor” OR “SGLT2” OR “sodium glucose cotransporter 2 inhibitors” OR “canagliflozin” OR “dapagliflozin” OR “empagliflozin” OR “ertugliflozin” OR “tofogliflozin” OR “bexagliflozin” OR “henagliflozin” OR “ipragliflozin” OR “licogliflozin” OR “luseogliflozin” OR “remogliflozin” OR “sergliflozin” OR “sotagliflozin”) AND TS ═ (“randomized controlled trial” OR “randomized” OR “randomly” OR “placebo” OR “control” OR “allocated” OR “RCT”) AND TS ═ (“liver” OR “hepatic”) AND TS ═ (“fat” OR “adipose” OR “adiposity” OR “lipid”)

## Data Availability

The data that support the findings of this study are available from the corresponding author upon reasonable request.
